# Histological Evidence of the Osseointegration of Fractured Direct Metal Laser Sintering Implants Retrieved after 5 Years of Function

**DOI:** 10.1155/2017/9732136

**Published:** 2017-08-27

**Authors:** Francesco Mangano, Carlo Mangano, Adriano Piattelli, Giovanna Iezzi

**Affiliations:** ^1^Department of Medicine and Surgery, Dental School, University of Varese, 21100 Varese, Italy; ^2^Department of Dental Sciences, Vita and Salute University S. Raffaele, 20132 Milan, Italy; ^3^Department of Oral and Biotechnological Science, University G. D'Annunzio, 66013 Chieti, Italy

## Abstract

**Background:**

Direct metal laser sintering (DMLS) is an additive manufacturing technique that allows the fabrication of dental implants layer by layer through the laser fusion of titanium microparticles. The surface of DMLS implants is characterized by a high open porosity with interconnected pores of different sizes; therefore, it has the potential to enhance and accelerate bone healing. To date, however, there are no histologic/histomorphometric studies in the literature evaluating the interface between bone and DMLS implants in the long-term.

**Purpose:**

To evaluate the interface between bone and DMLS implants retrieved after 5 years of functional loading.

**Methods:**

Two fractured DMLS implants were retrieved from the human jaws, using a 5 mm trephine bur. Both the implants were clinically stable and functioned regularly before fracture. The specimens were processed for histologic/histomorphometric evaluation; the bone-to-implant contact (BIC%) was calculated.

**Results:**

Compact, mature lamellar bone was found over most of the DMLS implants in close contact with the implant surface; the histomorphometric evaluation showed a mean BIC% of 66.1% (±4.5%).

**Conclusions:**

The present histologic/histomorphometric study showed that DMLS implants were well integrated in bone, after 5 years of loading, with the peri-implant bone undergoing continuous remodeling at the interface.

## 1. Introduction

Today, modern implant dentistry offers innovative surgical and prosthetic protocols such as the placement of implants in extraction sockets and immediate functional loading [1, 2]. These protocols are primarily aimed at meeting the patient's desires of fewer surgical sessions (with reduced invasiveness and stress and discomfort, resulting from multiple surgeries) and receiving their fixed prosthetic rehabilitation in the shortest time possible (to avoid the physical and psychological discomfort related to provisionalization with removable dentures, whether partial or total, and to reduce the time of treatment) [1–3].

Although these protocols represent an opportunity to reduce the costs related to implant-prosthetic treatment, they are simultaneously a challenge for the clinician who must ensure the survival/success of implants and related restorations in a context of greater difficulties and risks [3–5]. In fact, the placement of implants in extraction sockets can be risky given that it is more difficult for the surgeon to obtain a valid primary implant stabilization (the surgical alveolus is generally wider than the implant, and the stabilization must often be obtained apically in only 3-4 mm) [1, 3]. The primary stabilization may be even more complex in areas characterized by low bone density, as in the posterior maxilla [3, 5, 6]. The immediate functional prosthetic loading can represent a risk, as mechanical stress beyond a certain threshold may interfere with the healing process at the interface between bone and implant and thus with the osseointegration of the fixture [2, 4–6]. This risk is greater for single restorations placed in low bone density areas [5, 6], and may even lead to implant loss.

Recently, manufacturers have introduced a series of new micro- and nanorough implant surfaces to the market with the aim of enhancing bone healing, accelerating the time of prosthetic treatment, and reducing the risks arising from the application of modern surgical and prosthetic protocols [7–9]. These surfaces derive from particular treatments such as anodizing [7], acidification with hydrofluoric acid [8], and coating with calcium phosphate-nanoparticles [9].

Another innovative procedure for the fabrication of dental implants today is direct metal laser sintering (DMLS) [10, 11]. Such an additive manufacturing technique allows the creation of dental implants from micro- and nanotitanium powders that are fused together by a powerful laser beam [10, 11]. The implants are constructed layer by layer according to a computer-assisted design [11]. The surface resulting from this innovative fabrication technique is characterized by a high porosity with interconnected pores of different sizes [11–13].

To date, the best way to study the interface between the bone and the implant is represented by histological studies on humans [14].

Several histological studies have shown that, in the short term, the porous surface of DMLS implants can support an excellent bone healing [15–18]. Therefore, it is not surprising that these fixtures can be successfully used in complex surgical and prosthetic protocols, such as placement in extraction sockets and immediate functional loading [19, 20].

However, until now all human histologic/histomorphometric works on the interface between bone and the surface of DMLS implants were based on the evaluation of experimental (small-size) fixtures inserted in a transitional period (for example, to support a complete provisional removable prosthesis) and then removed for histological evaluation [15–18]. This is for ethical reasons, as it is not ethically acceptable to insert (and later be removed for histologic evaluation) implants of standard dimensions in man [15]. In addition, all histologic/histomorphometric works on DMLS implants available today have studied the interface between the bone and the implant surface in the short term, that is, a few months after placement of the fixtures [15, 17, 18]. It is clear that the early healing period immediately following implant placement is a delicate moment and therefore important to be investigated, as this can determine the success of the rehabilitation. However, an evaluation of osseointegration in the long-term can be extremely interesting because it can clarify much about the relationship that develops between the implant surface and human bone over time [21, 22].

To effectively evaluate the relationship between the implant surface and bone over time, we should be able to remove the implants after a fairly long period of function, possibly several years. This is rarely possible because most of the implants removed (for infection or progressive loss of bone) may not be used for this purpose [22]. The fracture of the implant body is fortunately a rare event, because it is a major complication for the clinician [21, 22]. However, it is an unlikely event that can be useful for the long-term evaluation of the interface between bone and implant in man [21, 22]. To date, there are no histological studies in the literature evaluating the interface between bone and DMLS implants of standard dimensions in the long-term [13].

Therefore, the purpose of the present work was to study the interface between bone and standard size DMLS implants in order to fully understand the dynamics that occur at that level in the long-term. For this purpose, we have histologically evaluated standard size DMLS implants, which were perfectly integrated into the bone but removed for fracture after 5 years of function.

## 2. Materials and Methods

### 2.1. Implant Fabrication and Surface Characteristics

**Figure 1:**
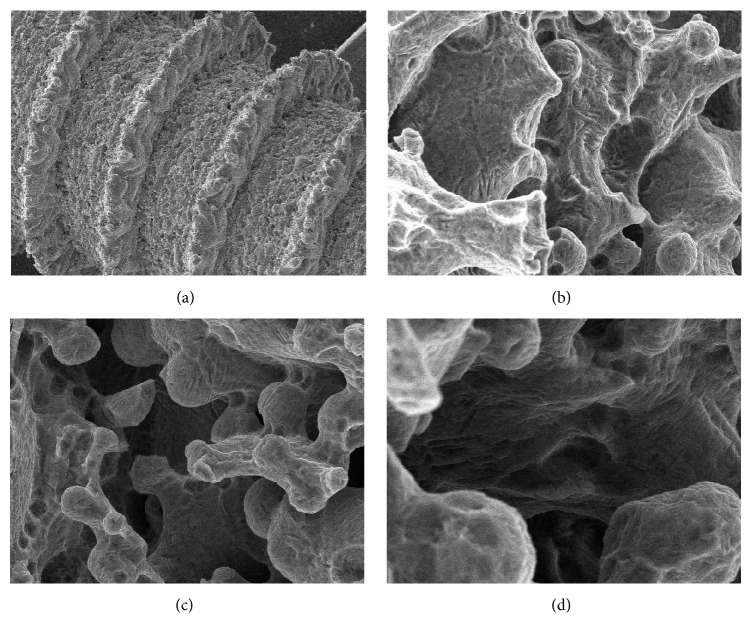
The scanning electron microscopy (SEM) evaluation of the DMLS titanium implant showed a porous surface ((a) magnification 47x) with globular protrusions ((b) magnification 842x), rich in cavities interconnected with by pores ((c) magnification 1100x), and irregular crevices ((d) magnification 2270x).

The DMLS fixtures (TixOs®, Leader Implants, Cinisello Balsamo, Italy) were fabricated from Ti-6Al-4V micropowders (diameter: 25–45 *μ*m). The implants were fabricated in ytterbium laser machine (Eosint270®, EOS, Munich, Germany) in an argon atmosphere. The laser had the possibility of constructing 250 × 250 × 215 mm of volume and used a wavelength of 1,054 nm with a power (continuous) of 200 W and a 7 m/s of scanning rate; the laser spot had a size of 0.1 mm. The residual weakly adherent particles of Ti-6Al-4V were removed as follows. The implants were sonicated in distilled water for 5 minutes at a temperature of 25°C and then were immersed in hydrogen peroxide (20 g/l) and NaOH (20 g/l) for 30 minutes at a temperature of 80°C. The implants were further sonicated in distilled water for other 5 minutes at a temperature of 25°C. Finally, the fixtures were cleaned by immersion in an organic acid mixture comprised of 50% maleic acid and 50% oxalic acid for 45 minutes at a temperature of 80°C and were washed in a sonic bath of distilled water for 5 minutes. Sterilization was obtained using gamma radiation, and then the implants were packed. The surface roughness of the DMLS had an *R*_*a*_ of 66.8 *μ*m, *R*_*q*_ of 77.55 *μ*m, and *R*_*z*_ of 358.3 *μ*m (Figure 1) as previously reported [11–13].

### 2.2. Implant Retrieval and Evaluation

Two DMLS titanium fixtures and the surrounding hard tissues were retrieved after fracture of the implant body occurred after 5 years of functional prosthetic loading. Both of these implants were located in the anterior regions (one in the anterior maxilla and the other in the anterior mandible) of two different patients (a 45-year-old and a 70-year-old man, resp.) where they supported a fixed implant-supported prosthesis and a removable overdenture, respectively. Both of these implants were stable before removal and did not suffer from any infection; the fixtures were removed using a 5 mm trephine bur.

### 2.3. Specimen Processing

The implants were retrieved after 5 years of prosthetic loading and processed as previously reported [14, 22] in order to perform histologic and histomorphometric evaluation. In brief, the specimens were first washed with saline and then immediately fixed using 0.1% glutaraldehyde and 4% paraformaldehyde in a 0.15 mol/L cacodylate buffer at pH of 7.4 with a temperature of 4°C. The specimens were processed for histology as follows. Thin sections were obtained with the aid of a dedicated cutting machine (Precise Automated One®, Assing Systems, Rome, Italy) and were dehydrated in a series of ascending alcohol rinses. These sections were then embedded in resin glycol methacrylate (Technovit 7200 VLC®, Heraeus, Wehrheim, Germany). After polymerization was completed, the specimens were cut using diamond disks and grinding machines to approximately 30 *μ*m. Acid fuchsin and toluidine blue were used to stain the slides, which were observed under a polarized-light microscope (Laborlux S®, Leitz, Wetzlar, Germany). Histomorphometry of the percentages of bone-to-implant contact was calculated by means of the aforementioned microscope connected to a camera with high resolution (JVC3CCDJVCKYF55B®, JVC, Yokohama, Japan) and interfaced to a monitor and personal computer (Pentium III 1200 MMX®, Intel, Santa Clara, CA, USA). A digitizing pad (D-Pad®, Matrix Vision, Oppenweiler, Germany) was associated with the optical system and a histometry software package with the capability to capture images (ImageProPlus4.5®, Immagini&C, Milan, Italy). For the histomorphometric evaluation, the bone-to-implant contact (BIC%), defined as the amount of mineralized bone in direct contact with the implant surface, was measured around all implant surfaces. Means and standard deviations of BIC% were calculated for each implant and then for all implants.

## 3. Results

**Figure 2:**
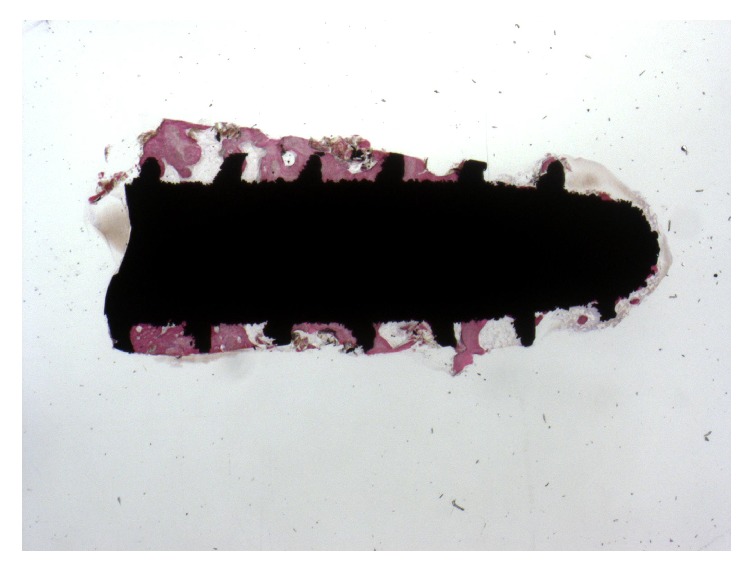
Specimen harvested from the anterior maxilla of a 45-year-old patient. Trabecular, mature bone at the interface of the implant. The first bone-to-implant contact was located at the level of the fracture line of the implant. Bone remodeling areas were present. Acid fuchsin-toluidine blue, magnification 12x.

**Figure 3:**
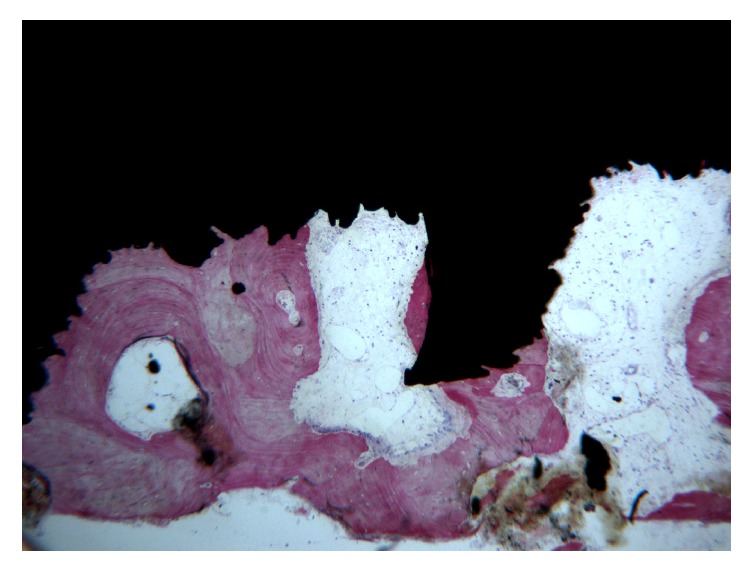
Bone remodeling areas and marrow spaces were present near the implant surface. No gaps were evident at the interface. A rim of osteoblasts making new osteoid matrix on the implant surface was evidenced. Acid fuchsin-toluidine blue, magnification 40x.

**Figure 4:**
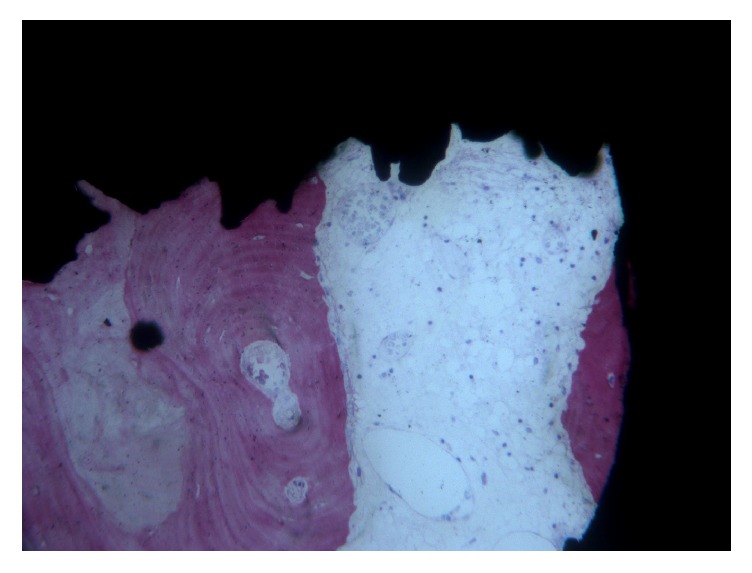
Blood vessels of different sizes were present within the marrow spaces. Osteoid matrix was evident inside the marrow spaces and secondary osteons could be seen abutting the implant surface. Acid fuchsin-toluidine blue, magnification 100x.

**Figure 5:**
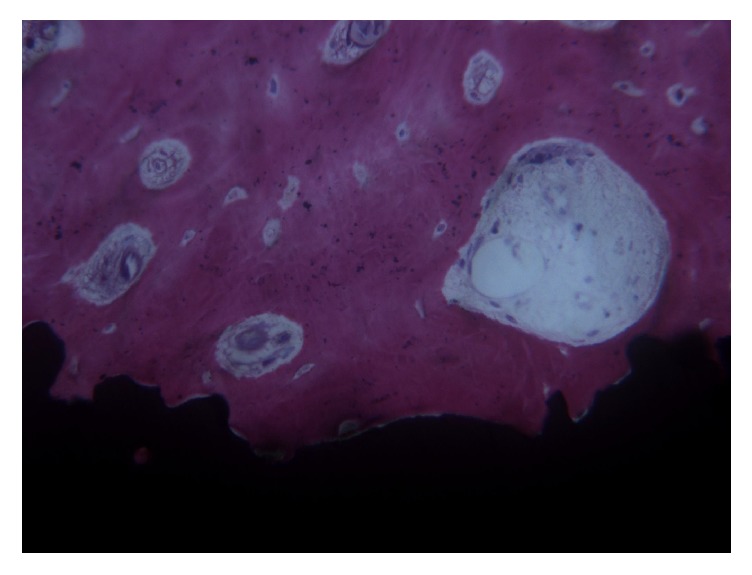
Bone was in tight contact with the implants surface and adapted to all its microirregularities. Acid fuchsin-toluidine blue, magnification 200x.

Bone trabeculae were evidenced around all the implants at low magnification. The first bone-to-implant contact was located at the level of the fracture line. In the apical portion of the interface, small amounts of newly formed bone in close contact to the implant surface could be observed (Figure 2). Around the implant, bone was present in different maturation stages and many remodeling areas and reversal lines were evident (Figure 3). Near the implant surface, small and large marrow spaces were detected with many blood vessels and osteoblasts present. Those osteoblasts appeared in the process of forming new bone starting from the implant surface toward the marrow space (Figure 4). At high magnification, bone was adapted to the microirregularities of the implant surface. In some areas of the peri-implant bone, it was possible to see osteocyte lacunae in close contact with the implant surface. No fibrous tissues or gaps were present at the interface (Figure 5). The histomorphometrical evaluation showed a mean bone-to-implant contact of 66.1% (±4.5%).

## 4. Discussion

In recent years, patients have become increasingly demanding, requiring minimally invasive treatments and a reduction of the number of surgical sessions and time of treatment [1–4, 19, 20]. In this sense, it is not surprising that the immediate implant placement in extraction sockets and immediate functional loading have great success today [2, 4, 19, 20]. The placement of implants in fresh extraction sockets immediately after the extraction of nonrestorable teeth allows clinicians to reduce the number of surgical sessions and the invasiveness of the treatment while decreasing inconvenience and psychological stress for the patient [1, 3, 19]. At the same time, the possibility of immediately loading the implants allows the industry to globally reduce the duration of implant-prosthetic treatment and restore aesthetics and function without periods of temporization with removable dentures which is usually unwelcome to the patient [2–4].

The establishment of new surgical (such as the placement of immediate implants in extraction sockets) [3, 19] and prosthetics (such as immediate loading) protocols [2, 4, 20] is now a clinical reality, but also a challenge for dentists as the survival and success of implant-supported rehabilitation must be obtained in a context of greater risks [2–4, 6]. It is well known that the stabilization of an implant in a fresh postextraction socket can be difficult [1, 3]. Similarly, the immediate functional loading can represent a risk for an implant because the prosthetic load may transmit micromovements at the bone-implant interface and these movements, if they exceed a certain threshold, can interfere with bone healing and osseointegration [2, 4–6].

In order to enhance the integration of the fixture in the bone to reduce healing time and anticipate the functionalization, a series of new implant surfaces with micro- and nanotopographical features have been recently introduced into the market with the aim of stimulating and promoting bone formation [7–9, 23].

An alternative solution to these surface treatments is now represented by three-dimensional (3D) printing or additive manufacturing techniques applied to the world of implantology [10, 11]. Direct metal laser sintering (DMLS) is an additive manufacturing technique that builds objects layer by layer starting from metal powders [10, 11]. This technique can be effectively used to fabricate dental implants through the fusion of titanium micropowders by means of a powerful laser beam. The fixtures are then fabricated layer by layer according to a computer-assisted-design (CAD) project [11–13, 15].

Several in vitro studies have demonstrated that titanium DMLS implants possess a highly porous surface structure with an open interconnected porosity where the surface concavities are connected with internal pores through a dense network of tunnels and interconnections [11–13, 24]. This honeycomb structure may be able to support the rapid formation of fibrin networks and drive the cell migration and the differentiation of the mesenchymal stem cells into functional osteoblasts capable of producing new bone [11–13].

Several histologic and histomorphometric studies have investigated the osseointegration of DMLS titanium implants in different animal models [25–28].

In a biomechanical and histologic/histomorphometric study on the beagle dogs, Witek et al. [25] compared the early bone response to laser sintered and alumina blasted/acid-etched implants. Four implants were placed in the radius of 18 Beagle dogs; after a period of 1, 3, and 6 weeks, all implants were retrieved for histologic/histomorphometric evaluation [25]. At the end of the study, a significantly higher BIC% was observed in the laser sintered implants only at 1 week, whereas no significant differences were reported for the two groups of implants, at 3 and 6 weeks, respectively [25]. However, the laser sintered implants showed biocompatible and osteoconductive properties and an improved biomechanical response with higher removal torque at 1 and 6 weeks, when compared to alumina blasted/acid-etched implants [25].

In another interesting histomorphometric and microCT study, Cohen et al. [26] compared the bone response to two different implants in the rabbit femur: laser sintered solid and porous implants. In this study, both microCT and histomorphometry showed significantly higher new bone volume for porous compared to solid implants, and bone growth was observed in porous implant pores, especially near apical portions of the implant [26]. Accordingly, the authors concluded that laser sintered implants with micro/nanoscale surface roughness and trabecular porosity can stimulate new bone growth and may therefore be used as a superior alternative to solid implants for bone-interfacing implants [26].

These results confirmed the evidence emerging from a previous in vitro study [27], in which the same authors suggested that a 3D architecture may enhance the osseointegration of dental implants in vivo.

In contrast, Bowers et al. [28] found no statistically significant difference in the BIC% of laser sintered and resorbable blasting media dental implants, installed in the mandible of six sheep, and retrieved after an undisturbed period of six weeks.

However, the human histologic/histomorphometric studies are certainly the best way to investigate the bone-implant interfaces [14, 22, 23].

Previous histologic and histomorphometric researches have investigated the interface between bone and DMLS implants in the first period of healing; however, those were experimental and transitional fixtures of reduced dimension removed 2 months after insertion [15–18, 24].

In a histologic/histomorphometric study on the human posterior maxilla (type IV bone), 30 transitional mini-implants (10 DMLS titanium implants, 10 machined implants, and 10 dual acid-etched implants) were inserted in 30 patients (one implant per patient) and left unloaded for a period of 2 months. After that, the fixtures were retrieved for histologic and histomorphometric examination [24]. The BIC% was higher for the DMLS and DAE implants compared with the machined implants (*p* = 0.0002) [24].

Similar results were found in a subsequent human histologic/histomorphometric study where 12 fully edentulous subjects had two DMLS experimental implants in the posterior maxilla installed. One fixture was immediately loaded, whereas the other was left unloaded [15]. The loaded implants supported a complete removable denture for 2 months [15]. After this period, the experimental implants and their surrounding tissue were retrieved and processed for histologic/histomorphometric analysis [15]. The authors found mature, woven, preexisting bone lined by new bone in the early maturation stages. The histomorphometric analysis showed a mean BIC% of 45.20% (±7.68%) and 34.10% (±7.85%) for immediately loaded and unloaded fixtures, respectively (*p* < 0.05) [15]. Therefore, the authors concluded that although both loaded and unloaded implants showed high percentage of bone contact, immediately loaded fixtures had a higher BIC% [15].

In another study, four patients were installed with experimental, transitional DMLS titanium implants [16]. The implants were installed in the posterior mandible and then retrieved with the surrounding tissues after 2 months in order to perform a scanning electron microscopy (SEM) and optical histologic analysis [16]. The SEM evaluation showed new bone with calcium and phosphorus, whereas the histometric evaluation found a mean BIC% of 60.5 ± 11.6% [16].

In another report, the same authors found a BIC% of 69.5% in experimental, transitional DMLS titanium implants placed in the posterior maxilla. These were left unloaded for a 2-month period and then retrieved for histologic evaluation [17]. In particular, the peri-implant bone was in tight contact with the surface, marrow spaces were evidenced in other areas, and the cement lines were prominently stained [17].

Our present histologic/histomorphometric work is the first that has examined the interface between bone and DMLS implants of standard size that underwent functional loading for a period of 5 years, and it seems to confirm the findings of the previous aforementioned reports. In fact, the histologic sections depicted trabecular, mature bone around the entire implant surface with many remodeling areas. Bone was in tight contact with the implant surface and adapted to all its microirregularities, and rims of osteoblasts depositing osteoid matrix directly on the implant surface could be observed. In accordance with the previous literature, a satisfactory high mean BIC% of 66.1% (±4.5%) was found. The present work confirmed that the 3D environment of cavities, tunnels, and pores of various dimensions obtained with the DMLS technique and the subsequent treatment of the surface with organic acids (oxalic and maleic acids) may provide an optimal substratum for bone tissue ingrowth after functional loading in the long-term.

It could be hypothesized that bone formation within the concavities of the DLMS surface occurs when mesenchymal stem cells migrate into the pores, stop proliferation, and start the differentiation into functional osteoblasts [12, 13]. In particular, the porous surface of DMLS implants may enhance the proliferation and differentiation of bone cells [29], while growth factors (for example, bone morphogenetic proteins) may become concentrated within cavities and then slowly be released over time [29].

In the face of these supposed biological advantages which must be confirmed by further studies, doubts emerge about the mechanical resistance of titanium implants fabricated with DMLS technology [30]. A fixture should not fracture after 5 years of function. From the clinical point of view, the fracture of the implant body is a major complication that forces the clinician to remove and replace the fixture with another one. This removal can be technically difficult and may result in a large bone defect [30], especially where integration with the hard tissues is optimal and the contact between bone and implant is so high.

## 5. Conclusions

In this present study, a histologic/histomorphometric evaluation of the peri-implant tissues around two fractured DMLS titanium implants removed from the human mandible after 5 years of functional loading was performed. Bone appeared consistently adherent to the surface, as revealed by the light optical microscopy. The hard tissue grew into the concavities of the titanium surface and completely filled the implant threads. The DMLS implants appeared well integrated over the long-term, with bone tissue around the implant undergoing continuous remodeling. In conclusion, the present study confirms that the DMLS surface may provide an excellent substratum bone tissue ingrowth after functional loading in the long-term. However, controlled histologic/histomorphometric studies are needed to further validate the present results.
